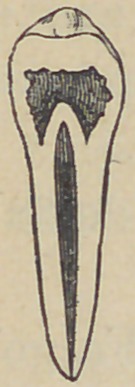# A Singular Case

**Published:** 1885-01

**Authors:** 


### A Singular Case.

The following peculiar case was presented recently in the practice of Dr. G. R. Thomas, of Detroit. The peculiarities and features are so unusual as to render it well worthy of record. A young man, about twenty years of age, presented himself for examination of his teeth; all of which, after careful examination, were pronounced perfectly sound. On the gum, opposite the root of the second superior bicuspid, on the right side, there was present what seemed to be a small, superficial aphtheous ulcer. It had given little or no pain or inconvenience. It was touched with tincture of iodine, supposing that within a day or so it would pass away. On being presented again two or three days afterward, it was found to be much larger and much deeper: it was again touched and the patient dismissed, and again presented two or three days afterward. The affection had increased, showing enlargement and a more aggravated appearance. Within it was a hard protuberance, which, upon being examined, was supposed to be a fragment of Alveolus. This was removed with a sharp engine burr, after which the patient was dismissed. Upon returning again within a few days, the root of the tooth seemed to be quite involved in irritation, and, indeed, actual disease. Examination was now made with a view of ascertaining whether the pulp of the tooth was living, which revealed almost to a certainty that it was dead. In order to further determine the condition of the tooth, the pulp chamber was opened by drilling through the fissure upon the masticating surface. The drill, after passing a little way, sank suddenly into what seemed to be a large cavity, much larger than the normal pulp chamber. Exploration of this cavity showed that the walls were lined with a soft material. The pulp canal into the root of the tooth could not be entered; it seemed obstructed or closed by a projection or prominence of some hard material. A small right angle excavator was introduced, and with considerable effort was pushed through the cement on the palatine side. It broke down a thin wall and passed to the alveolus; then broaches, both rigid and flexible, were used, but entrance to the canal could not be effected. A complete exploration of the cavity in the crown forced to the conclusion that it could not be filled, and that, in fact, the tooth was of no special value. Its extraction, therefore, was decided upon. This was done, and afterward the tooth was split completely through longitudinally, in order to ascertain its exact condition—which is shown by the accompanying cut.

The cavity in the crown was twice the capacity of the normal pulp chamber, with its walls much softened, communication between this cavity and the canal in the root was completely shut off by a cone of dentine, built up and protruding by a sharp point into this crown cavity; it also formed a complete and acutely arched roof over the root canal. The contents of the cavity in the crown, when first opened, was of an offensive, vitiated character, very similar to the debris of dead pulp. The contents of the root canal consisted of the debris of the dead pulp of that part. How long this had been dead it is difficult to determine. That the living pulp in the root should form for itself a covering and protection against the debris of dead pulp in the crown is certainly very remarkable. The condition of the pulp in the crown and the disintegration of the dentine are also very strange. It is true that occasionally, under unusual irritation, the pulp produces the absorption of a small amount of dentine in its chambers, but in no case, so far as record shows, at least, has so extensive a disintegration been effected as in this instance. That this should be done, and that the pulp in the root canal should become so devitilized and decayed and produce no periostial disturbance is another remarkable circumstance of this case. The fact that the pulp formed such an effectual shield for itself against the offensive matter in the crown is an illustration of the great activity and the reparative power of this organ. It teaches an important lesson in this respect. This case is one of unusual interest, and well illustrates the value of a thorough investigation of every unusual case presented. Has anybody ever seen anything analogous to it?

## Figures and Tables

**Figure f1:**